# Supplementary material for: Quantitative Analysis of Plasma Cell-Free DNA and Its DNA Integrity and Hypomethylation Status as Biomarkers for Tumor Burden and Disease Progression in Patients with Metastatic Neuroendocrine Neoplasias
Source: Cancers (Basel). 2022 Feb 17;14(4):1025. doi: 10.3390/cancers14041025 (PMC8870292; doi:10.3390/cancers14041025)
Supplement: Supplementary file 1 [file cancers-14-01025-s001.zip › cancers-1532030-supplementary.pdf]

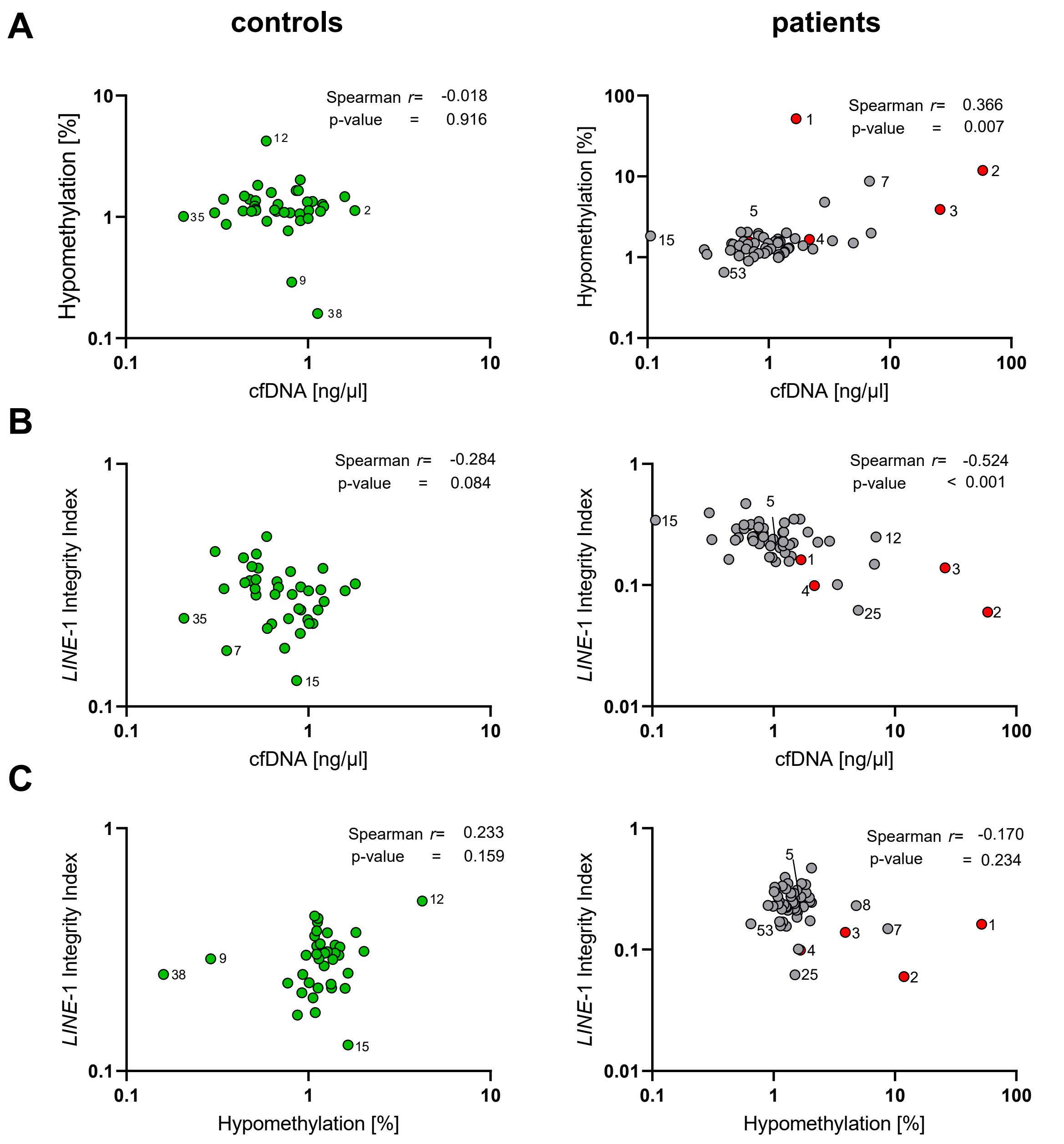

Figure S1. Scatter Plot and Spearman correlation analysis for (**A**) cfDNA concentration and *Alu*-hypomethylation, (**B**) cfDNA concentration and *LINE*-1 integrity, and (**C**) *Alu* hypomethylation and *LINE*-1 integrity of controls (green) and patients (grey). Deceased patients are marked in red color and outliers are tagged for identification. r= Spearman's correlation coefficient.

Table S1: Results of Spearman Correlation analysis of cfDNA characteristics with respect to age and gender, tumor differentiation, tumor grade, proliferation (Ki67-index), and current tumor treatment (watch and wait, SSA, PRRT, chemotherapy, immunotherapy, external radiation, and combination treatment).

**controls (n=38)**				
		cfDNA concentration	*Alu* hypomethylation	*LINE-1* integrity
age	Spearman's rho	0.237	0.036	-0.214
	p-value	0.153	0.828	0.198
gender	Spearman's rho	0.237	-0.179	-0.116
	p-value	0.153	0.283	0.488

**patients (n=53)**				
		cfDNA concentration	*Alu* hypomethylation	*LINE*-1 integrity
age	Spearman's rho	0.216	-0.145	0.075
	p-value	0.12	0.3	0.596
gender	Spearman's rho	-0.021	0.025	-0.016
	p-value	0.881	0.861	0.909
differentiation	Spearman's rho	0.047	0.086	-0.133
	p-value	0.736	0.542	0.342
tumor garde	Spearman's rho	0.073	0.195	-0.151
	p-value	0.612	0.171	0.291
ki67 [%]	Spearman's rho	0.13	0.308	-0.011
	p-value	0.444	0.064	0.95
treatment	Spearman's rho	-0.077	0.166	-0.134
	p-value	0.581	0.234	0.338

Table S2: Sensitivities and specificities of the ROC analysis of plasma cfDNA concentration, *Alu* hypomethylation, and *LINE-1* integrity with respect to tumor burden as shown in Figure 5.

		**healthy controls vs. patients**	**cured patients vs. patients**
**cfDNA concentration**			
low TB	sensitivity %	54.5	40.9
	specificity %	58.6	88.9
	cut off [pg/µl]	>0.78	>0.92
moderate TB	sensitivity %	78.3	65.2
	specificity %	58.6	88.9
	cut off [pg/µl]	>0.75	>0.93
high TB	sensitivity %	75	87.5
	specificity %	100	77.8
	cut off [pg/µl]	>1.64	>0.9
***Alu*-hypomethylation**			
low TB	sensitivity %	59	68.2
	specificity %	51.72	88.9
	cut off %	>1.235	>1.15
moderate TB	sensitivity %	73.9	86.9
	specificity %	51.72	88.9
	cut off %	>1.245	>1.135
high TB	sensitivity %	100	100
	specificity %	82.76	100
	cut off %	>1.595	>1.5
***LINE-1* integrity**			
low TB	sensitivity %	77.3	86.4
	specificity %	55.2	44.4
	cut off	<0.293	<0.319
moderate TB	sensitivity %	73.9	73.9
	specificity %	62.07	55.6
	cut off	<0.281	<0.282
high TB	sensitivity %	87.5	87.5
	specificity %	75.9	66.7
	cut off	<0.231	<0.240

Table S3: Multiparametric ROC analysis using cfDNA concentration and/or percentage of Alu-hypomethylation and/or LINE-1 integrity to estimate the strength of the model to discriminate between healthy controls or cured patients and NEN patients with low, moderate, and high tumor burden, with area under the curve (AUC), p-value, negative predictive power (NPP) [%], and positive predictive power (PPP) [%]. n.a.: not analyzed since not defined for the chosen cut-off of > 0.5.

**tumor burden**		**controls vs. patients**			**cured patients vs. patients**		
		low	moderate	high	low	moderate	high
***Alu* hypomethylation + *LINE-1* integrity**	AUC	0.661	0.727	0.944	0.859	0.928	n.a.
	p	0.05	0.0053	0.0001	0.002	0.0002	n.a.
	NPP	62.86	70	93.33	62.5	70	n.a.
	PPP	56.25	63.64	85.71	82.61	90.9	n.a.
***cfDNA concentration + LINE-1* integrity**	AUC	0.627	0.784	0.849	0.566	0.749	0.861
	p	0.123	0.0005	0.0028	0	0.0309	0.0124
	NPP	58.97	72.41	90.63	n.a.	60	100
	PPP	50	65.22	100	70.97	77.78	81.82
cfDNA concentration + Alu hypomethylation	AUC	0.577	0.762	0.884	0.818	0.889	n.a.
	p	0.351	0.0013	0.001	0.006	0.0007	n.a.
	NPP	59.57	67.65	93.55	66.7	70	n.a.
	PPP	75	66.67	100	80	90.9	n.a.
cfDNA concentration + Alu hypomethylation + LINE-1 integrity	AUC	0.666	0.795	0.931	0.864	0.918	n.a.
	p	0.0438	0.0003	0.0002	0.0017	0.0003	n.a.
	NPP	61.76	73.33	93.55	66.67	75	n.a.
	PPP	52.94	68.18	100	86.36	87.5	n.a.
